# Comparison of FLASH Proton Entrance and the Spread-Out Bragg Peak Dose Regions in the Sparing of Mouse Intestinal Crypts and in a Pancreatic Tumor Model

**DOI:** 10.3390/cancers13164244

**Published:** 2021-08-23

**Authors:** Michele M. Kim, Ioannis I. Verginadis, Denisa Goia, Allison Haertter, Khayrullo Shoniyozov, Wei Zou, Amit Maity, Theresa M. Busch, James M. Metz, Keith A. Cengel, Lei Dong, Costas Koumenis, Eric S. Diffenderfer

**Affiliations:** Department of Radiation Oncology, Perelman School of Medicine, University of Pennsylvania, Philadelphia, PA 19104, USA; Michele.kim@pennmedicine.upenn.edu (M.M.K.); vioannis@pennmedicine.upenn.edu (I.I.V.); denisa.goia@pennmedicine.upenn.edu (D.G.); allison.haertter@pennmedicine.upenn.edu (A.H.); khayrullo.shoniyozov@pennmedicine.upenn.edu (K.S.); wei.zou@pennmedicine.upenn.edu (W.Z.); amit.maity@pennmedicine.upenn.edu (A.M.); theresa.busch@pennmedicine.upenn.edu (T.M.B.); James.Metz@pennmedicine.upenn.edu (J.M.M.); keith.cengel@pennmedicine.upenn.edu (K.A.C.); lei.dong@pennmedicine.upenn.edu (L.D.); costas.koumenis@pennmedicine.upenn.edu (C.K.)

**Keywords:** proton FLASH radiation, spread-out Bragg peak

## Abstract

**Simple Summary:**

FLASH radiotherapy is a treatment technique of interest that involves radiation delivered at ultra-high dose rates >100 times faster than traditional radiation therapy, which has been shown to spare radiation damage to normal tissue but maintain tumor control capabilities. Proton therapy uses spread-out proton Bragg peaks to reduce radiation dose to normal tissue by directing the highest dose of radiation to the tumor volume. In this study, irradiation of the whole abdomen of mice was performed with proton beams at FLASH dose rates in order to investigate the normal tissue sparing capabilities of the spread-out Bragg peak compared to the entrance region of the proton depth dose curve.

**Abstract:**

Ultra-high dose rate FLASH proton radiotherapy (F-PRT) has been shown to reduce normal tissue toxicity compared to standard dose rate proton radiotherapy (S-PRT) in experiments using the entrance portion of the proton depth dose profile, while proton therapy uses a spread-out Bragg peak (SOBP) with unknown effects on FLASH toxicity sparing. To investigate, the biological effects of F-PRT using an SOBP and the entrance region were compared to S-PRT in mouse intestine. In this study, 8–10-week-old C57BL/6J mice underwent 15 Gy (absorbed dose) whole abdomen irradiation in four groups: (1) SOBP F-PRT, (2) SOBP S-PRT, (3) entrance F-PRT, and (4) entrance S-PRT. Mice were injected with EdU 3.5 days after irradiation, and jejunum segments were harvested and preserved. EdU-positive proliferating cells and regenerated intestinal crypts were quantified. The SOBP had a modulation (width) of 2.5 cm from the proximal to distal 90%. Dose rates with a SOBP for F-PRT or S-PRT were 108.2 ± 8.3 Gy/s or 0.82 ± 0.14 Gy/s, respectively. In the entrance region, dose rates were 107.1 ± 15.2 Gy/s and 0.83 ± 0.19 Gy/s, respectively. Both entrance and SOBP F-PRT preserved a significantly higher number of EdU + /crypt cells and percentage of regenerated crypts compared to S-PRT. Moreover, tumor growth studies showed no difference between SOBP and entrance for either of the treatment modalities.

## 1. Introduction

FLASH radiotherapy (RT) involves the delivery of ultra-high dose rates, above approximately 40 Gy/s [1,2], to elicit normal tissue sparing while maintaining the tumor cell killing capabilities of conventional dose rates below 1 Gy/s. Several studies have observed FLASH effects (i.e., enhanced normal tissue protection) with electrons [1,2,3,4,5]. FLASH RT has recently been investigated with proton RT (PRT) with clinically available cyclotrons [6,7,8,9,10] and synchrocyclotrons [11,12], investigating mouse whole abdomen irradiation, leg contracture, and skin toxicity [6,13]. Proton RT, compared to photon and electron RT, offers enhanced localized dose delivery and increased relative biological effectiveness in the proton Bragg peak [14]; however, the largest draw to proton RT is the reduced exit dose through the utilization of the Bragg peak [15]. To date, proton FLASH radiobiology studies have used a shoot-through method irradiating with the entrance region of a high-energy proton beam and delivering the Bragg peak beyond the target (typically outside of a patient/mouse). The ionization density and linear energy transfer (LET) of the proton dose is increased in the Bragg peak, leading to an enhancement of relative biological effect [14,16]. To the best of our knowledge, the impact of LET increase on the proton FLASH sparing effect has not been reported and this is the first demonstration of the toxicity sparing effects of proton FLASH on normal tissues using the spread-out Bragg peak (SOBP) region of proton dose deposition.

Beamline design limitations present a challenge to investigating the FLASH effect in the Bragg peak region. Pencil beam scanning (PBS) proton beams, which have been widely implemented for clinical treatments at conventional dose rates, are created by delivering sequential layers of pristine Bragg peaks to individual spots (on a millimeter scale) via quadrupole magnet steering to an entire three-dimensional target volume. Cyclotron produced protons for PBS require energy degraders along the beamline to reduce the incident proton energy in order to ensure that proton ranges are appropriate to deliver Bragg peaks to all distal and proximal layers in the target. These energy degraders cause a large decrease in efficiency, with beam losses up to 99% for 70 MeV protons [17], which greatly reduces the dose rate. Beam loss along with other challenges and unknowns have been discussed elsewhere [17,18,19].

Kourkafas et al. recently reported the creation of a single scattering system with a rotating modulator wheel to create a proton SOBP at FLASH dose rates of approximately 75 Gy/s [20]. However, no biological studies were conducted to demonstrate the FLASH effect at the SOBP. Electron studies suggest that higher dose rates (exceeding 100 Gy/s) could hold an enhanced normal tissue sparing effect beyond that observed for lower FLASH range dose rates [2]. An alternative method for SOBP creation that does not require the added time structure considerations of a modulator wheel and motor is a ridge filter, and FLASH dose rates in a proton SOBP have been demonstrated with a ridge filter using a clinical cyclotron [10] and synchrocyclotron [12].

Ridge filters are proton beam-forming devices similar to modulator wheels in that they take the monoenergetic proton beam that impinges it, and with varying material thicknesses, create a polyenergetic proton beam that will have varying ranges and Bragg peak locations. The combination of these varying Bragg peak locations will then form a larger, fixed plateau width SOBP [21,22]. However, unlike modulator wheels, ridge filters are stationary devices placed along the beamline; thus, they require no added time considerations. By using a stationary ridge filter to define the SOBP width, variable dose rates are achievable for a fixed proton beam energy, allowing for quick and seamless transitions between FLASH and conventional dose rates. In this study, we demonstrate the capability and film validation of SOBP proton delivery to targets at FLASH dose rates through a double-scattering system with energy modulation performed by a ridge filter.

## 2. Materials and Methods

### 2.1. Proton Delivery and SOBP Generation

**Figure 1:**
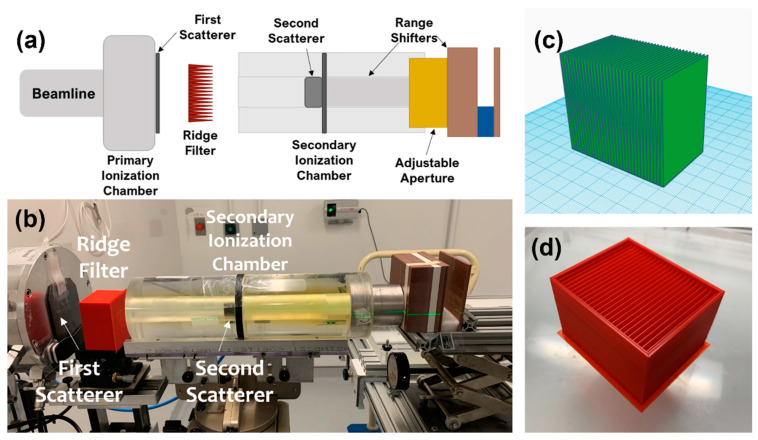
(**a**) Schematic and (**b**) photo of the beam line design with ridge filter and range shifters to deliver a spread-out Bragg peak (SOBP) in the mouse. (**c**) Model of ridge filter and (**d**) photo of the printed ridge filter with protective casing.

An IBA Proteus Plus with a C230 cyclotron (IBA Proton Therapy, Louvain-La-Neuve, Belgium) was used to deliver a 230 MeV (range ~32 g/cm^2^) beam on the fixed angle beam line in the dedicated research room. Beam alignment and beam control were described previously [7,23]. Briefly, a pulse generator was used to modulate proton beam current via the beam current regulation unit (BCREU) of the IBA control system. The dose was set by stopping the current pulse to the BCREU with the output of a preset counter connected to an electrometer measuring charge from a transmission ionization chamber (primary ionization chamber in Figure 1a at the end of the beam line). The time width of each proton beam pulse time was recorded to determine the dose rate. Previous work has demonstrated a linear relationship between the measured dose and preset counts at FLASH dose rates, indicating sufficient response time in the electronic system for the short FLASH beams [7]. The beam current from the cyclotron was set between 2 nA and 360 nA in order to achieve the desired dose rate at the target.

A ridge filter was designed and printed using a 3D printer (X-Max, Zhejiang QIDI Technology Co., Ltd., Ruian, Zhejiang, China), as shown in Figure 1c,d. The final printed version included a protective case for reproducible placement and ease of handling. For this study, all mice were irradiated with the 230 MeV proton beam. Range shifters were included in the beam line to deposit the SOBP in the mice. Dose rate was varied while maintaining the set-up geometry by changing the cyclotron current. The ridge filter had dimensions of 40 mm (depth) × 64 mm (length) × 56 mm (height) and was comprised of 32 individual ridges. Each ridge was made of a triangle shape with a base width of 2 mm.

### 2.2. Proton Beam Dosimetry

Absolute dosimetry was performed with a calibrated NIST traceable Advanced Markus Chamber (PTW, Freiburg, Germany) applying corrections for temperature, pressure, polarity effects, and recombination according to the International Atomic Energy Agency Code of Practice TRS-398 [24]. A thin window Bragg peak chamber (Type 34070, PTW, Freiburg, Germany) placed in the beam line (see the secondary ionization chamber in Figure 1a,b) was cross-calibrated with the absolute dose measurements prior to each experiment in order to perform online dosimetry. Absorbed dose is reported with no correction for relative biological effect (RBE) difference of protons relative to X-rays or between the entrance and SOBP regions of the depth dose profile.

Recombination of the Advanced Markus Chamber used for absolute dosimetry was investigated previously [7,9,25]. In the papers by Diffenderfer et al. [7] and Yin et al. [25], a charge-collecting Faraday cup [26] (which does not suffer from recombination effects) was used to characterize the Markus chamber. The chamber showed no evidence of signal loss at the FLASH dose rates used in this study for bias voltages greater than 200 V. Recently, a two- and three-voltage technique was used to derive ion recombination correction factors for the Markus chamber and showed no significant difference from the theoretically derived values calculated from saturation curves [25].

Lateral beam profiles within the SOBP were measured with radiochromic film (Gafchromic EBT3, Ashland, Bridgewater, NJ, USA) placed between 2–10 mm layers of solid water plastic (Gammex, Sun Nuclear, Melbourne, FL, USA) for the full range of the SOBP. The relative dose at each film depth was measured. The depth profile was measured with a multi-layer ionization chamber (Zebra, IBA Dosimetry, Louvain-La-Neuve, Belgium). The LET of the proton beam is relatively low in the entrance portion of the depth dose profile at ~0.4 keV/μm, rising to ~2–3 keV/μm for the mid-SOBP irradiations [27,28,29].

Irradiated film was scanned on an Epson 1000XL flatbed scanner (Epson America Inc., Long Beach, CA, USA) using 300 dpi scan settings. Monoenergetic 230 MeV protons were used to generate a SOBP with a ridge filter, resulting in various energy protons at the SOBP. Film was used as a measure of relative dose (comparing only high-LET-irradiated film) and normalizing to the measured SOBP from a multi-layer ionization chamber. The absolute dose was measured using an Advanced Markus chamber placed in the mid-SOBP. The response of the film with the red channel was used to derive relative depth dose characteristics in the high-LET SOBP.

### 2.3. Murine Studies

Eight- to ten-week-old female C57BL/6J mice (The Jackson Laboratory, Bar Harbor, ME, USA) were maintained in the University of Pennsylvania Association for Assessment and Accreditation of Laboratory Animals Care (AAALAC)-accredited animal facilities. All experimental procedures were conducted in accordance with protocols approved by the Institutional Animal Care and Use Committee. Mice were checked daily and euthanized upon onset of severe morbidity, including hunched posture, social withdrawal, relative immobility, or apparent weight loss >20%. For the proton irradiation studies, 2% isoflurane in medical air was used to anesthetize mice. Mice were euthanized by CO_2_ asphyxiation, and small intestine (jejunum) segments were harvested and preserved in optimal cutting temperature (OCT) medium.

Mice were randomly placed into one of five experimental groups: (1) FLASH dose rate SOBP, (2) standard dose rate SOBP, (3) FLASH entrance region, (4) standard entrance region, and (5) non-irradiated controls. Healthy mice received irradiation to the whole abdomen at a dose of 15 Gy using a 2 × 2 cm^2^ field size. Table 1 summarizes the groups and their respective dose rates and doses. The doses and dose rates reported were along the central axis of the lateral beam profile. For the SOBP studies, mid-SOBP doses and dose rates are reported. Mice were placed with the beam entering from the right flank and exiting on the left flank with the whole abdomen (bottom of lung to top of pelvis) positioned so that it was centered in the 2 × 2 cm field. The lateral width of the mice was small enough to completely fit in the 2.5-cm width holder for the SOBP studies.

In the tumor studies, 5 × 10^5^ MH641905 mouse pancreatic tumor cells (syngeneic to C57Bl/6 mice) [7] were injected into the flanks of 8- to 10-week-old C57BL/6J mice. At ten days post-injection (median tumor volume 150–170 mm^3^), mice were randomly assigned to receive a single dose of 18 Gy with FLASH or standard proton radiotherapy at the entrance region or SOBP with a focal beam (1 cm diameter). Tumors were measured with calipers 3–4 times per week, and mice were euthanized when tumor volume surpassed 1500 mm^3^. The doses and dose rates for these studies are reported in Table 1.

### 2.4. EdU Proliferation Assay

At 3.5 days post-irradiation (IR), all mice were injected intraperitoneally (i.p.) with 200 mg of 5-ethynyl-20-deoxyuridine (EdU; Thermo Fisher Scientific, Waltham, MA, USA; Cat: C10337) in phosphate buffer saline 2–3 h before euthanasia, as described previously [30]. EdU is incorporated into newly synthesized DNA and is a measure of cell proliferation. EdU was detected according to the manufacturer’s instructions. Number of EdU+ cells/crypt and % of regenerated crypts were assessed by counting at least 100 crypts per mouse section. Data were derived from three biologically independent experiments. Microscopy was performed on the Zeiss Observer. Z1 (Jena, Germany).

### 2.5. Statistical Analysis

Statistical analysis was conducted using GraphPad Prism 9.1.2 software (San Diego, CA, USA). For EdU analysis, an unpaired two-tailed Student’s *t* test was used. Survival data were summarized with Kaplan–Meier methods and tested using the Log-rank (Mantel–Cox) test. The alpha value was set at 0.05. Data presented as mean ± standard error of the mean, *n* ≥ 3/group in all experiments.

## 3. Results

**Figure 2:**
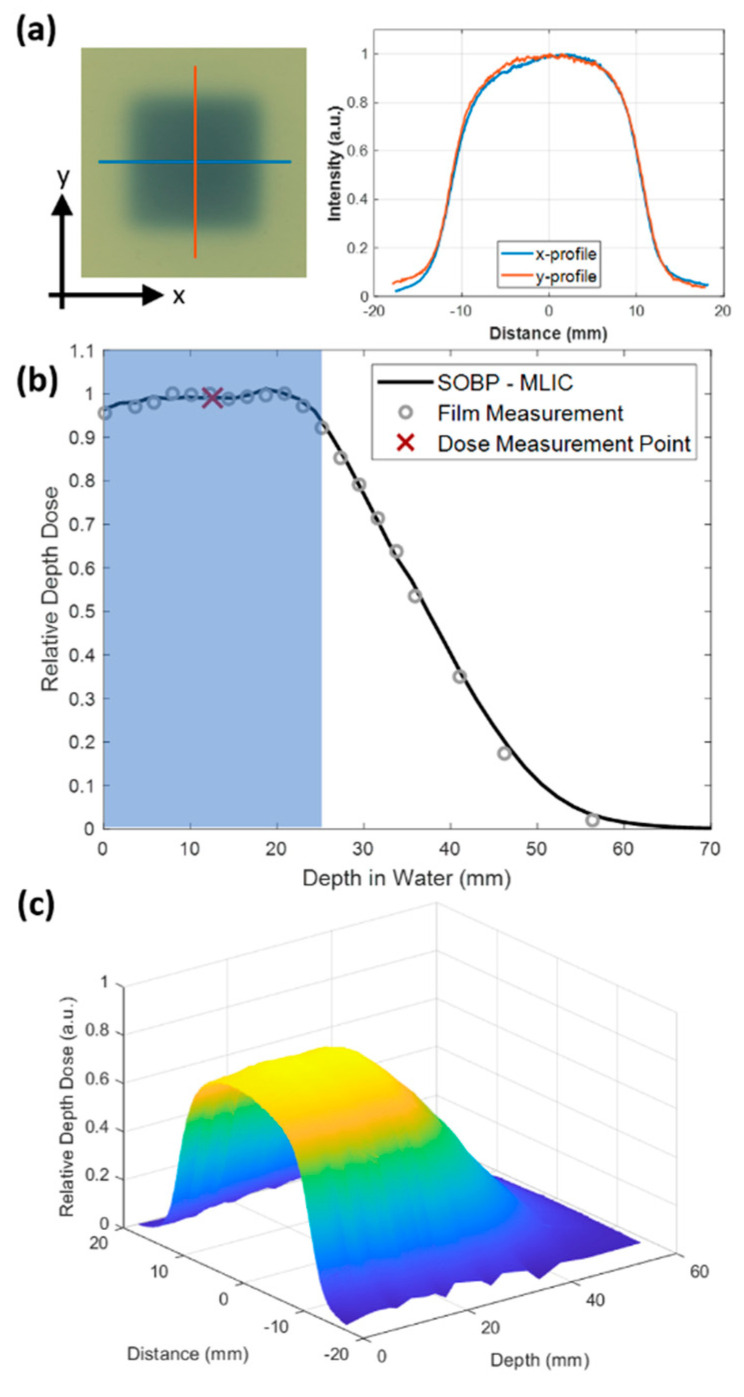
(**a**) EBT3 Gafchromic film irradiated at the mid-SOBP with a 2 × 2 cm^2^ field size. x and y lateral profiles are shown on the right. (**b**) Relative depth dose of SOBP used to irradiate mice measured by the multi-layer ionization chamber (MLIC). Mice were placed in a holder in order to be within the shaded blue region. Film (circles) and multi-layer ion chamber measurements (solid line) agreed. The red x indicates the point at which absolute dose measurements were performed for the SOBP studies. (**c**) Surface plot of relative depth dose measured by a stack of 20 films with 2 to10 mm pieces of solid water plastic between films.

A square collimated field size of 2 × 2 cm^2^ was achieved at both the entrance and the SOBP using the double-scattering system (Figure 2a). The resulting SOBP had a modulation (width) of 2.5 cm to the distal 90% of the maximum dose (Figure 2b). The measured SOBP from the multi-layer ionization chamber agreed with the measured SOBP from the stacked radiochromic film after normalization (Figure 2b). Mice were irradiated uniformly across the whole abdomen using the entrance of the high-energy proton beam or with the SOBP. Stacked film profiles were acquired and used to verify field size across the modulation of the SOBP, as seen in Figure 2c. Range-shifting material placed in the beam line allowed for the SOBP to be delivered to the whole mouse abdomen while using the high-energy proton beam.

**Figure 3:**
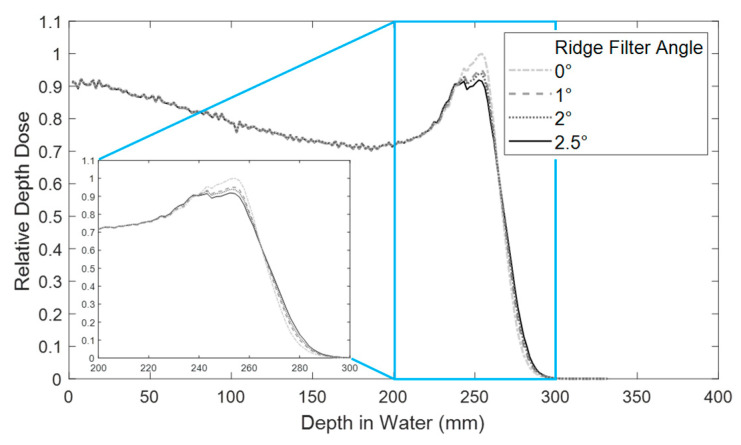
Relative depth dose measurement with a ridge filter in the beam line at various angles. The inset plot shows a zoom of the SOBP region. All studies were performed with the ridge filter at 2.5° for a flat SOBP.

SOBP modulation was altered depending on the angle of the ridge filter with respect to the beam axis (Figure 3). Due to the beam profile shape after the first scatterer and the design of the ridge filter with triangular peaks, the angle of the ridge filter affected the flatness of the SOBP modulation. A 2.5° angle was chosen for all experiments in order to obtain a flat SOBP modulation.

The average dose rate for the FLASH-irradiated mice was 108 ± 12.3 Gy/s, and the average dose rate for the standard-irradiated mice was 0.82 ± 0.16 Gy/s. With the lowest current achievable from the cyclotron (2 nA), the dose rate was found to be 0.82 ± 0.14 Gy/s at the SOBP. The mice irradiated in the entrance region were matched to have a similar dose rate (0.83 ± 0.19 Gy/s) with a slightly higher cyclotron current (3 nA). FLASH dose rates at the SOBP and entrance region were 108 ± 8.3 Gy/s and 107 ± 15.3 Gy/s, respectively.

**Figure 4:**
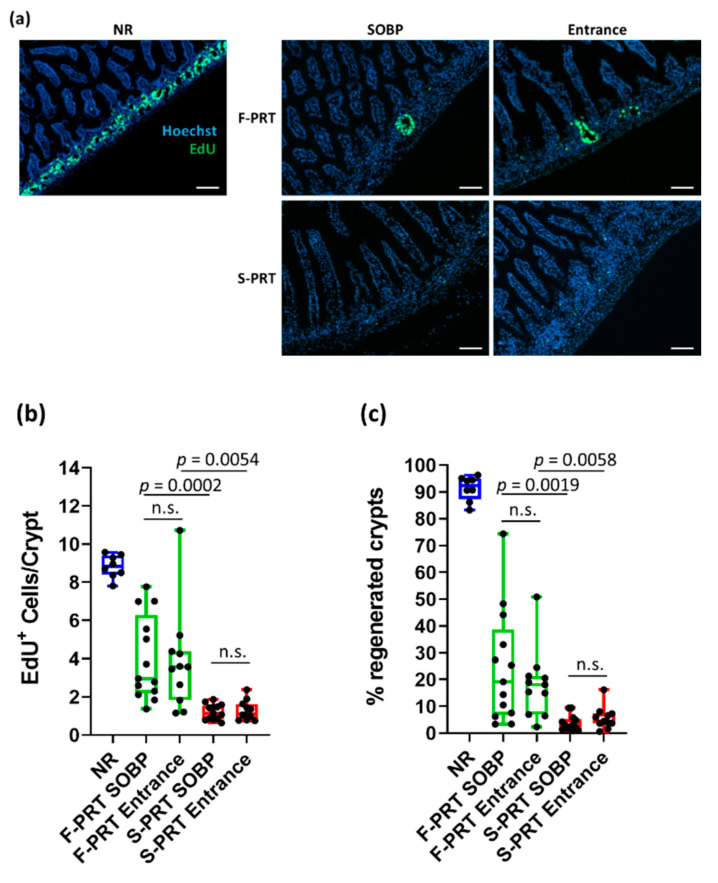
(**a**) Representative images of EdU (green) staining in frozen jejunum sections at 3.5 days post 15 Gy of whole abdominal irradiation (10× magnification; scale bar 100 mm) with FLASH proton radiotherapy (F-PRT) or standard proton radiotherapy (S-PRT) dose rate protons in the entrance and SOBP region of the depth dose profile. (**b**) Quantification of EdU+ cells per crypt. (**c**) Quantification of the % regenerated crypts. Data presented as mean ± SEM; n.s. = not significant.

Analysis of EdU incorporation showed that FLASH proton radiotherapy (F-PRT) SOBP normal tissue-irradiated mice showed a significantly higher number of EdU+ cells per crypt and a higher percentage of regenerated crypts than S-PRT SOBP, although both were significantly decreased compared with the non-irradiated tissues (NR) (Figure 4a–c). Interestingly, no significant difference in EdU+ cells and regenerated crypts was observed between SOBP and shoot-through method in both treatment modalities (Figure 4a–c).

**Figure 5:**
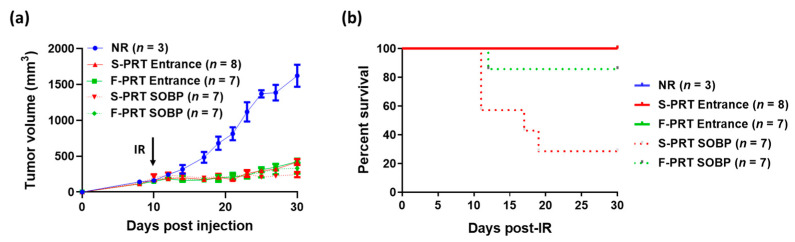
(**a**) Tumor growth curves of mice following 18 Gy focal irradiation with protons at standard (S-PRT Entrance, solid red; S-PRT SOBP, dotted red) versus FLASH (F-PRT Entrance, solid green; F-PRT SOBP, dotted green) dose rates. Unirradiated mice served as a control group (NR, blue). Black arrow indicates the time of irradiation. Data expressed as mean ± SEM. (**b**) Kaplan–Meier survival analysis of the mice from (**a**).

To further evaluate the effect of SOBP and shoot-through methods in both of the proton RT treatment modalities on tumor growth, we used a mouse pancreatic flank tumor model [7]. All RT treatment modalities presented very similar tumor growth inhibition post focal irradiation of 18 Gy (Figure 5a). There was a trend (albeit not a significant difference) towards increased tumor control with the SOBP (both standard and FLASH), which we attribute to the increased relative biological effect (RBE), which is recognized as being 1.0 in the entrance region and 1.1 in the SOBP relative to high-energy photons [14]. Moreover, survival analysis on tumor-bearing mice revealed that although all the S-PRT and F-PRT entrance-treated mice survived the treatment, approximately 70% of the S-PRT SOBP-treated mice succumbed to radiation lethality within the first 20 days post-RT. Interestingly, only 15% of the F-PRT SOBP mice died post-irradiation (Figure 5b) (*p* = 0.0371 vs. S-PRT SOBP).

## 4. Discussion

This study represents the first biological evidence of normal tissue sparing using the SOBP for FLASH proton radiotherapy while demonstrating equipotent tumor growth control using the two proton RT modalities. While both entrance and SOBP F-PRT showed a significantly higher number of EdU+/crypt cells and percentage of regenerated crypts compared to S-PRT, the results are also suggestive of increased tumor control with the SOBP relative to the entrance dose for both S-PRT and F-PRT. Additionally, survival analysis of the tumor-bearing mice revealed that significantly more mice died of SOBP S-PRT compared to SOBP F-PRT while maintaining equivalent tumor control in the two cohorts. The preservation of the stem cell compartment in the intestinal crypts post-irradiation is critical for the maintenance of a functional intestinal barrier. The proliferation status of the stem/progenitor crypt cells is an indication of their functionality. Previous studies have shown that the EdU assay (proliferation assay) labels the proliferative cells and serves as an effective marker of acute gastrointestinal toxicity prediction [7,30,31].

Prior studies with proton FLASH have been with the low-LET entrance region of the proton depth dose curve using a shoot-through technique [6,7,8,9]. There is a clinical advantage to using the higher LET spread-out Bragg peak for proton treatment due to the increased capability of achieving conformal dose distributions with fewer beams by using the Bragg peak where a higher LET is observed. This study indicates the independence of the FLASH effect on the relative LET found in therapeutic proton SOBPs. Given the apparent dependence of a FLASH effect on dose rate, maintaining a conformal dose with 1 or 2 proton SOBP beams is desirable. Furthermore, the evidence points to the higher LET found in the SOBP improving tumor control while reducing toxicity or lethality when using F-PRT over S-PRT. Biological evidence of the FLASH effect with an SOBP will enable further development of highly conformal tumor-targeted FLASH radiotherapy.

While there is some dose rate fluctuation within the FLASH-irradiated group, no significant biological difference was observed in that range with dose rates above 90 Gy/s within this study. Dose rates varied due to inherent cyclotron current fluctuations and experiments being performed on different days; however, the total dose delivered was consistent, with <1% variation.

Current pencil beam scanning systems were evaluated for potential FLASH delivery capabilities by our group (Zou et al.) [18]. With cyclotron output currents above 500 nA, the effective single energy field FLASH dose rate that is above 40 Gy/s was found to only be achievable with fields less than 4 × 4 cm^2^. Energy switching time will be the limiting factor for achieving high dose rates for a field. By using a single energy and single field with a ridge filter, larger fields at higher dose rates may be achievable in a clinically relevant setting.

Further implementation and expansion of studies using the proton SOPB in FLASH mode are currently underway to address issues related to tumor control as well as differences seen with increased LET within the SOBP.

## 5. Conclusions

SOBP FLASH proton beams were delivered to the whole abdomen of healthy mice. FLASH-irradiated mice exhibited normal tissue sparing and similar tumor growth control compared to that achieved with standard dose rate proton radiotherapy. Use of the SOBP for proton FLASH can lead to better dose conformality around targets and increased normal tissue sparing.

## Figures and Tables

**Table 1:** Experiment summary with doses and dose rates expressed as average ± standard deviation for each group.

Group	Tissue	Number of Mice	Dose Rate (Gy/s)	Dose (Gy)
SOBP FLASH	Normal whole abdomen	18	108.2 ± 8.3	15.0 ± 0.1
SOBP Standard		18	0.82 ± 0.1	15.0 ± 0.04
Entrance FLASH		20	107.1 ± 15.3	15.0 ± 0.1
Entrance Standard		18	0.83 ± 0.2	15.0 ± 0.1
Control		5	--	--
SOBP FLASH	Tumor	7	106.2 ± 0.5	18.0 ± 0.1
SOBP Standard		7	0.70 ± 0.01	18.1 ± 0.02
Entrance FLASH		7	118.5 ± 0.4	18.0 ± 0.02
Entrance Standard		8	0.74 ± 0.003	18.0 ± 0.1
Control		3	--	--

## Data Availability

The data presented in this study are available on request from the corresponding author.
